# Mother-to-child transmission of HIV: findings from an Early Infant Diagnosis program in Bertoua, Eastern Cameroon

**DOI:** 10.11604/pamj.2013.15.65.2551

**Published:** 2013-06-21

**Authors:** Jean Jacques N Noubiap, Adamo Bongoe, Sylvie Agokeng Demanou

**Affiliations:** 1 Internal Medicine Unit, Edéa Regional Hospital, Edéa, Cameroon; 2 Gyneco-obstetric Unit, Edéa Regional Hospital, Edéa, Cameroon; 3 Laboratory, Bertoua Regional Hospital, Bertoua, Cameroon

**Keywords:** Mother-to-child transmission of HIV, early infant diagnosis, polymerase chain reaction, Cameroon

## Abstract

**Introduction:**

Early diagnosis of HIV is crucial to ensure early antiretroviral (ARV) treatment which is associated with lower mortality in HIV-infected children. This study reports the prevalence of HIV infection and the factors associated to mother-to-child transmission (MTCT) in an Early Infant Diagnosis (EID) program in Bertoua, Cameroon.

**Methods:**

We reviewed the records of 112 HIV-exposed infants aged six weeks to 18 months who had an HIV-1 DNA PCR test done in 2010. Data included socio-demographic characteristics, clinical manifestations of HIV, ARV prophylaxis, feeding options and results of the PCR tests.

**Results:**

The median age at first HIV testing was 4 months (IQR, 2-7). Ninety-one point one percent of infants and 65.2% of mothers did not receive ARV prophylaxis. Fifty infants (44.6%) were exclusively breastfed, 37 (33%) received formula feeding and 25 (22.4%) received mixed feeding. The prevalence of HIV in the infants was 11.6%. MTCT of HIV was significantly associated with mixed feeding (adjusted odds ratio (aOR): 6.7, 95% CI 1.6-28.3; p=0.009) and an age at 1st PCR test greater than 6 months (aOR: 6.5, 95% CI 1.4-29.3; p=0.014). The mothers of 66.1% of the infants tested returned to collect the result.

**Conclusion:**

There is a high rate of MTCT of HIV in this setting, due to a poor implementation of the PMTCT program. There is a critical need to increase the use of ARV prophylaxis, and to improve rapid first testing and completion of the EID. The infant feeding practices also have to be improved.

## Introduction

Mother-to-child transmission (MTCT) of HIV resulted in approximately 370,000 infant infections worldwide in 2009 [1]. This same year, an estimated 2.5 million children worldwide were living with HIV, mostly a consequence of MTCT, and more than 90% of these children are in sub-Saharan Africa [1]. In the absence of any intervention, the combined risk of MTCT of HIV in utero and intra-partum is 15-30%. The risk is increased in breastfed children to 20-45% [2, 3]. It has been proven that antiretroviral (ARV) drugs given to HIV-positive pregnant women and their newborn babies reduce the risk of MTCT [4–6]. In resource-limited countries, about 35% and 52% of HIV infected infants without any therapeutic intervention die by age one and two respectively [7]. Observational studies in developed countries and a randomized clinical trial in South Africa demonstrated that early initiation of Highly Active Anti-Retroviral Therapy (HAART) in infants considerably reduces morbidity and mortality [8–11]. Consequently, since 2010, the World Health Organization (WHO) recommends systematic early initiation of HAART to all HIV-infected children diagnosed within the first two years of life, irrespective of CD4 count or WHO clinical stage [12, 13]. Early Infant Diagnosis (EID) of HIV infection is therefore crucial to achieve this recommendation.

In infants born from HIV-infected women, maternal anti-HIV antibodies cross the placenta and persist in infant blood for up to 18 months. These antibodies usually represent exposure to maternal HIV rather than true infant HIV infection [14, 15]. Therefore, HIV infection cannot be diagnosed in infants less than 18 months by antibody-based tests [14]. Only virological tests (which detect viral components in the blood) can be used for an accurate diagnosis of HIV before 18 months of age. The WHO estimates that in 2008-2009, only 6-15% of HIV-exposed infants benefited from recommended virological tests within the first year of life [16].

Since 2005, as part of prevention of mother-to-child transmission of HIV (PMTCT) interventions, many resource-limited countries are increasingly developing program of EID [17–20]. In 2007 the Cameroonian government launched an EID program in some health facilities [21]. A nationwide evaluation has reported a 12.3% overall prevalence of HIV-1 in infants born of HIV-positive mothers using the HIV-1 DNA PCR test [22]. The Bertoua Regional Hospital (BRH) was one of the two health facilities included in the East Region of Cameroon from the beginning of the program. This is the referral hospital of all the East Region of Cameroon which is the second most HIV-affected region in the country with an HIV prevalence of 8.6% in the adult population, and 11% among pregnant women [23]. PMTCT and antiretroviral therapy (ART) programs are routinely implemented in the BRH. This study aimed at reporting the prevalence of HIV infection and the factors associated with MTCT among the infants who had benefited from EID in 2010 in the BRH.

## Methods

### Study design and population

This is a retrospective analysis of data of all HIV-exposed infants enrolled in the EID program in the BRH from January to December 2010. After mothers’ counselling, HIV-exposed infants were referred for EID from PMTCT/Mother and Child health clinic and from AIDS Treatment Centre. From all the children eligible, Dried blood spots (DBS; whole blood obtained via heel stick or finger prick and dried on filter paper) were collected and couriered to a central laboratory at the Chantal Biya International Reference Centre for Research on HIV and AIDS Prevention and Management (CIRCB) for a HIV-1 DNA PCR test. The test was carried out using the Amplicor HIV-1 DNA PCR assay (Roche Diagnostics, Branchburg, NJ), which targets the HIV-1 *gag gene*. The test procedure has been described elsewhere [22]. Thirty days after samples were sent, PCR test results were sent back to the BRH where they were reported in a registry and made available to physicians. Caregivers of children with a positive HIV result were counselled to enrol them into HIV/AIDS Care and Treatment programs. According to the testing algorithm for EID in Cameroon, for all the children whose first test is positive, a second sample should be collected for confirmation four weeks after the first sample. A second test should also be done for breastfed infants, six weeks after weaning. A child is declared HIV-infected if the confirmation test is positive. Unfortunately, the majority of infants tested positive at the first test were not retested for confirmation because of their failure to attend the follow-up or to collect the result. We present in this study results of the first PCR tests.

### Data collection and analysis

This study was based on a review of data routinely collected in the EID register of the BRH's laboratory. Data included socio-demographic characteristics, clinical manifestations of HIV, type of chemoprophylaxis received by the mother and the baby, feeding options reported at the time of the first PCR test and results of the PCR tests. The proportion of parents/guardians receiving PCR test results was also noted.

Data were coded, entered and analysed using the Statistical Package for Social Science (SPSS) version 20.0. We described continuous variables using means with standard deviations or median with inter-quartile range (IQR), and categorical variables using their frequencies and percentages. The crude (unadjusted) correlates of mother-to-child transmission of HIV were examined in univariate logistic regression analysis. Multivariate forward stepwise logistic regression analysis was then done to identify independent factors with consideration for confounding effects and interactions. A p-value less than 0.05 was considered statistically significant.

Since all interventions within the EID program in the BRH were implemented under the supervision of the Regional office of the Ministry of Public Health of Cameroon and this study is based on existing data from EID register of BRH, the authors did not deem it necessary to seek approval from the National Ethics Committee of Cameroon.

## Results

During the study period, 125 HIV-exposed infants underwent an HIV-1 DNA PCR test. Of these, 112 were included in the analysis and 13 infants were excluded from the study for incomplete data.

Sixty-three (56.2%) infants were male and 49 (43.8%) were female. The median age at the time of HIV testing was 4 months (Inter-quartile range 2 to 7). The mean age of mothers was 28±6.2 years. Ninety-one point one percent (102/112) of infants and 65.2% (73/112) of mothers did not receive any ARV for PMTCT. Exclusive breastfeeding was the most common feeding option, present in 50/112 (44.6%) infants. Among the 80 infants aged 6 months or less, 40 (50%) were exclusively breastfed, 30 (37.5%) received formula and 10 (12.5%) received mixed feeding. Other baseline characteristics of infant/mother pairs are depicted in Table 1.

**Table 1:** Characteristics of the study population (N=112)

Characteristics	Number (%)
**Gender**	
Female	49 (43.8)
Male	63 (56.2)
**Age at 1** ^**st**^ **PCR test**	
≤ 6 weeks	13 (11.6)
> 6 weeks - 6 months	67 (59.8)
> 6 months -18 months	32 (28.6)
**Infant ARV**	
Single dose NVP at birth & AZT for 4 weeks	8 (7.1)
AZT + 3TC for 7 days	2 (1.8)
None	102 (91.1)
**Maternal ARV**	
HAART	2 (1.8)
AZT + 3TC & single dose NVP in labour	5 (4.5)
AZT & single dose NVP in labour	1 (0.9)
AZT + 3TC	2 (1.8)
Single dose NVP in labour	29 (25.9)
None	73 (65.2)
**Feeding option**	
Exclusive breastfeeding	50 (44.6)
Replacement feeding	37 (33)
Mixed feeding	25 (22.4)
**Cotrimoxazole prophylaxis in infant**	
Yes	12 (10.7)
No	100 (89.3)
**Clinical manifestations of HIV in infant**	
Yes	17 (15.2)
No	95 (84.8)

ARV: antiretroviral drugs; AZT: Zidovudine; 3TC: Lamivudine; NVP: Nevirapine

Based on the data of the first PCR tests, thirteen of the 112 infants were infected by HIV, giving an overall prevalence of 11.6%. As shown in Table 2, MTCT of HIV was significantly associated with mixed feeding (aOR: 6.7, 95% CI 1.6-28.3; p=0.009) and an age at 1st PCR test greater than 6 months (aOR: 6.5, 95% CI 1.4-29.3; p=0.014). The association between the absence of ARV prophylaxis in both mother and baby and MTCT of HIV was of borderline significance (aOR: 8.6, 95% CI 0.9-80.1; p=0.057). The mothers of 66.1% (N=74) of the 112 infants tested returned to collect the results.

**Table 2:** Correlates of mother-to-child transmission of HIV in the study population (N=112)

Characteristics	Total	HIV positive N (%)	Univariate analysis		Multivariate analysis	
			OR (95% CI)	P-values	aOR (95% CI)	P-values
**Gender**						
Female	49	3 (6.1)	Reference			
Male	63	10 (15.9)	2.8 (0.6 - 14.2)	0.11		
**Age at 1** ^**st**^ **PCR test**						
≤ 6 months	80	3 (3.7)	Reference		Reference	
> 6 months	32	10 (31.2)	11.6 (2.4 – 59.2)	<0.001	6.5 (1.4 – 29.3)	0.014
**Neither mother nor infant received ARV**						
No	44	1 (2.2)	Reference		Reference	
Yes	68	12 (17.6)	9.2 (1.16– 196.9)	0.01	8.6 (0.9 – 80.1)	0.057
**Feeding option**						
Exclusive breastfeeding or Replacement feeding	87	4 (4.6)	Reference		Reference	
Mixed feeding	25	9 (36)	11.6 (2.8 – 52.3)	<0.001	6.7 (1.6 – 28.3)	0.009
**Clinical manifestations of HIV in infant**						
No	95	10(10.5)	Reference			
Yes	17	3 (17.6)	1.8 (0.3 – 8.5)	0.31		

OR: unadjusted odds ratio; aOR: adjusted odds ratio

## Discussion

Early initiation of HAART in infants dramatically reduces HIV associated morbidity and mortality [8–11]. Indeed, the WHO pediatric treatment guidelines now recommend ART initiation for all HIV-infected infants under 24 months of age [12, 14]. EID is crucial for an early ART initiation. The WHO thus recommends HIV diagnostic testing for all HIV-exposed infants (infants born from HIV-infected mothers) at 4-6 weeks of age [12]. Unfortunately only 11.6% of infants in our study had been tested at 6 weeks of age or before. Moreover, the median age at first testing was 4 months in our study and higher than the 1.5 months found by Tejiokem et al. in Yaoundé and Douala [24]. This lower age at HIV testing found by Tejiokem et al. is a consequence of a very early enrolment in the EID process. In fact, the median age at enrolment of the HIV-exposed infants was 3 days (IQR, 1-5) in their study. After their enrolment few days after birth, samples collection from HIV-exposed infants for HIV testing was planned at 6 weeks of age. This process of EID was not well implemented in our setting. This difference in EID process could be explained by the fact that Tejiokem et al. conducted their study in three referral hospitals of the two largest cities of Cameroon. These referral hospitals are among the pioneers in the fight against HIV infection in Cameroon and offer a full range of services for infant and adult health needs in their areas [24]. PMTCT interventions are certainly more effective in these hospitals than in the BRH. This can be depicted by the fact that while 91.1% of infants and 65.2% of mothers did not receive any ARV prophylaxis for PMTCT in our setting, in the study of Tejiokem et al., only 9.9% of mother-baby pairs did not take any ARV prophylaxis for PMTCT [24]. Several of our mother-baby pairs never underwent any PMTCT interventions, and these infants were referred for EID because they presented clinical manifestations of HIV infections, resulting in delayed EID process. Moreover, the HIV/AIDS stigma is probably higher in semi-urban and rural areas such as Bertoua than in urban areas such as Douala and Yaoundé and may play a significant role in HIV infected mothers’ behaviour. This may influence their adherence to PMTCT interventions and could explain the higher MTCT rate found in our setting.

Inadequate PMTCT interventions result in high MTCT rates. Nkenfou at al. previously reported a MTCT rate of 12.69% in the East Region of Cameroon [22]. Similarly, our study shows a very high MTCT rate (11.2%) in Bertoua compared to the 3.6% found by Tejiokem et al. in Yaoundé and Douala [24]. This significant difference (p<0.001) between these two MTCT rates may be attributed to the ineffectiveness of PMTCT interventions in our setting. In Tanzania Nuwagaba-Biribonwoha et al. found a HIV prevalence of 17% among HIV-exposed infants in a setting experiencing significant difficulties in their PMTCT program [20].

The proportion of infants exclusively breastfed was higher in our study (44.6%) compared to the 10.7% found by Tejiokem et al. in Yaoundé and Douala [24]. Compared to women in urban areas such as Yaoundé and Douala, the lower economic status and higher socio-cultural pressure on feeding practices of women in Bertoua which is a semi-urban area could explain the fact that they are more prone to exclusively breastfeed their babies. The infant feeding policy in Cameroon is in accordance with the WHO guidelines on HIV and infant feeding which recommend that provided the mother and/or baby is receiving ARVs for their health or as prophylaxis, exclusive breastfeeding should be practiced by HIV-infected mothers for the first six months of life. After the six months period, complimentary feeding should be introduced while continuing with breastfeeding till 12 months of age unless replacement feeding is acceptable, feasible, affordable, sustainable and safe for them and their infants before that time [25]. In our study, among the infants aged 6 months or less, only 50.6% of them were exclusively breastfed, indicating an incomplete achievement of the WHO guidelines on HIV and infant feeding and thus emphasizing the need to improve infant feeding guidance among HIV positive mothers in Bertoua. We also found that 22.4% of infants in our study received mixed feeding. Oladokun et al. shown that in the African context, the major challenge faced by non-breastfeeding mothers is stigmatisation [26]. For the mothers who decide to give exclusive formula feeding to their babies, this stigmatisation can push them to mixed-feed their babies.

We found that HIV infection was significantly associated with an age at testing more than six months (p=0.014) and mixed feeding (p=0.009), suggesting that prolonged exposure to breastfeeding and particularly mixed feeding is likely to have affected HIV transmission rates for infants taking the DNA PCR test at a later date compared with those who had the test before 6 months. These findings are consistent with those of Illif et al. in Zimbabwe and Anoje et al. in Nigeria [27, 28]. Indeed, mixed feeding by HIV-infected mother, when compared to exclusive breastfeeding and replacement feeding has been shown to be associated with an increased risk of HIV transmission [29]. Exclusive breastfeeding may promote maintenance of the integrity of the infant's gastrointestinal barrier, which is thought to be the primary mode of infection. In addition, the immunological factors in breast milk likely reduce viral activity in human milk. Mixed feeding increases the risk of HIV transmission because the beneficial immune factors of breast milk are probably counteracted by the damage to the infant's gut wall by contaminants or allergens in mixed feeds [30]. Studies demonstrated that the absence of ARV prophylaxis in both mother and baby significantly increases MTCT rates [20, 23, 28]. This association between the absence of ARV prophylaxis in both mother and baby and MTCT of HIV was confirmed in our study by univariate logistic regression analysis (p=0.01), although the association in multivariate logistic regression analysis was of borderline significance (p=0.057).

There was no significant association between the presence of clinical manifestation of HIV and HIV PCR test positivity (p=0.3). This finding highlights the fact that the diagnosis of HIV in infants cannot efficiently rely on clinical manifestations. Although clinical and immunologic criteria can be used for presumptive diagnosis of HIV infection for the purpose of ART initiation [31], they have low sensitivity and specificity [32, 33] and clinical manifestations of HIV infection can be difficult to distinguish from those of other prevalent conditions in uninfected children such as malnutrition and tuberculosis [32].

In their study conducted in Yaoundé and Douala, Tejiokem et al. reported that 94.9% of the mothers of infants tested returned to collect the results [24], this proportion was 66.1% in our setting. Several EID programs in resource-limited settings have also experienced similar low proportions of EID results returned to the families/caregivers [19, 29, 34–37]. In our setting, this low proportion may have been partly due to a high rate of early mortality among the infants who never returned, possibly due to severe HIV infection. Other reasons include poor implementation of PMTCT, and poor awareness and understanding of parents who are dominated by HIV/AIDS stigmatisation.

One of the major impediments to the implementation of PMTCT in our setting is be the fact that a great number of pregnant women in this area do not attend antenatal clinic, tend to deliver their babies out of health facilities and thus have no or poor compliance to the PMTCT program. It is therefore crucial to improve public awareness on the importance of antenatal consultations and PMTCT interventions for pregnant women. There is also a need of more effective counselling of the mothers already enrolled in the PMTCT program in other to improve the precocity of HIV PCR testing and the completeness of the EID process.

One of the limitations of this study is the reduced number of infants included in our analysis. The study findings are also limited by the lack of testing confirmation for infants tested positive at the first PCR test because of their failure to attend the follow-up or to collect the result in most cases. However, this study which is one of the rare ones on PMTCT all over the country provides some relevant new insights on MTCT in the Eastern Cameroon. Further studies are needed to evaluate all the key steps in the cascade of EID and care process in BRH and in other sites in Cameroon, in order to identify the local obstacles that have to be overcome to achieve effective PMTCT and HIV care programs.

## Conclusion

The findings of this study demonstrate the weakness of PMTCT program in the BRH and probably in all the East Region of Cameroon, with consequence, a high rate of MTCT. There is a critical need to increase the uptake of ARV chemoprophylaxis, to improve the precocity of HIV PCR testing and the completeness of the EID process. The infant feeding practices also have to be improved.
